# Emergence of Exopolysaccharides Overproducers Is Linked to Environmental Spatial Structure via Redox State

**DOI:** 10.1128/msphere.00123-23

**Published:** 2023-04-10

**Authors:** Maxime Ardré, Maxime Deforet

**Affiliations:** a Laboratoire Biophysique et Evolution, CBI, ESPCI Paris, Université PSL, CNRS, Paris, France; b Sorbonne Université, CNRS, Institut de Biologie Paris-Seine (IBPS), Laboratoire Jean Perrin (LJP), Paris

**Keywords:** spatial structure, adaptation, cellular redox status

## Abstract

The formation of biofilm at the air-liquid interface of a still flask is related to the emergence of exopolysaccharides (EPS) overproducers. These mutants have the ability to remain near the surface, where oxygen is abundant. Yet, it is still unclear what role oxygen plays in cellular metabolism under this condition. A. Besse, M.-C. Groleau and E. Déziel (mSphere e00057-23, 2023; https://doi.org/10.1128/msphere.00057-23) explains that the redox state of cells is key in understanding the emergence of EPS overproducers. They found that the spatial distribution of oxidizing agent (not oxygen specifically) controls the advantage of remaining near the air-liquid interface, and hence the advantage that EPS-overproduction confers. All together this research paves the way for a deeper comprehension of the relationship between the environment's spatial structure and population dynamics.

## COMMENTARY

A bacterial culture flask left on the bench for many days will result in the production of biofilm at the air-liquid interface (ALI). A study by Besse and colleagues reveals with clarity that the seemingly simple observation has profound implications (1).

The formation of bacterial biofilm at the ALI is frequently triggered by the colonization of the surface by a mutant derived from the ancestral population. This mutant evolved to overproduce a polymer, which provides a competitive advantage at the ALI. Several mutants of exopolysaccharides (EPS) overproduction have been identified across species (2–4), as well as the metabolic pathways and mutations associated with them. They are mediated by diguanylate cyclases (DGCs), which regulate the secondary signaling molecule cyclic di-GMP (c-di-GMP) (5). Specifically, the mutant develops a mutation in the DGCs that regulate the amount of c-di-GMP, leading to an overexpression of c-di-GMP (4, 6) and subsequent polymer production. In a dynamic and spatially organized environment, these mutations are essential for survival.

**FIG 1:**
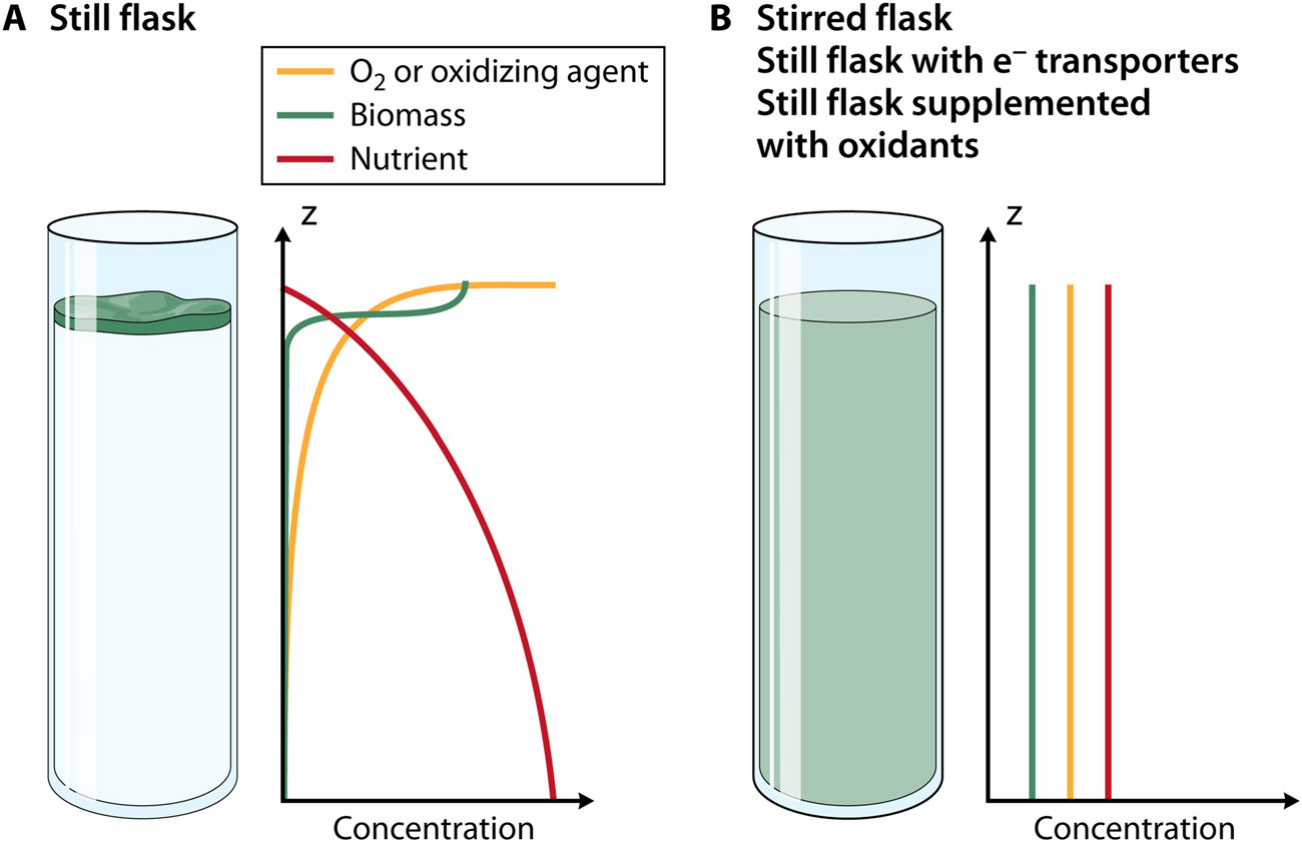
A: A still flask induces a spatial structure of O_2_ (the only oxidizing agent present in the media), which promotes the emergence of EPS-overproducing mutants and the formation of a biofilm at the air-liquid interface. B: When the flask is well stirred, or when the oxidizing agent is dispersed in the media (nitrate or pyocyanin), the spatial structure is suppressed and no biofilm forms at the air-liquid interface.

The abundance of oxygen at the ALI is generally accepted as the reason for colonization of this environment (7, 8). Fast use of oxygen by the bacterial population leads to an oxygen depletion in the bulk of the liquid: a significant gradient of oxygen rapidly develops in a still flask with virtually no oxygen 1 mm below the ALI (9) (Fig. 1A).

In this context, the ability for bacterial cells to remain at the liquid surface is critical. The insightful research undertaken by Besse et al. provides a clear description of the cellular metabolic state in relation to the oxygen availability.

The authors went beyond the notion that dissolved oxygen is the driving force behind biofilm development and demonstrated that, instead, any oxidizing agent could play a role in biofilm formation. While oxygen is the most common oxidizing agent, an alternate agent, such as nitrate, can be used to maintain a healthy metabolism. To prove their point, they measured the cells' metabolic activity in a flask by measuring their redox state through the NADH/NAD+ ratio. When nitrate is supplied to the bacterial population in place of oxygen for oxidation, much fewer EPS-overproducing mutants were found to invade the ancestral population (Fig. 1B). On the other hand, the authors ensured that a modified strain Δ*anr* that is incapable of metabolizing nitrate (due to the shutdown of the denitrification pathway by ANR) resulted in the selection of EPS-overproducing mutants even when nitrate was supplied. Moreover, they calculated the quantitative threshold of the NADH/NAD+ ratio below which the redox state of the population promotes the invasion of EPS-overproducing mutants.

## A FEEDBACK LOOP BETWEEN HOW THE OXIDIZING AGENT IS SPREAD AND HOW THE ALI BIOFILM IS MADE

Their research emphasizes that the spatial heterogeneity of an oxidizing agent is responsible for the selection of overproducer mutants at the ALI. Further evidence is shown when cultures are treated with pyocyanin, a diffuse metabolite that can transport electrons from the cell to a distant oxidant. Addition of pyocyanin to the flask makes the oxidizing agent's activity available across a greater distance. Therefore, the oxidizing environment becomes more homogeneous (less spatially structured, Fig. 1B), decreasing the advantage of remaining at the ALI. Mutants that produce excessive EPS are less likely to outperform the ancestral strain. Conversely, adding DNase reduced extracellular DNA, a known electron shuttle in biofilms (10), reinforced the spatial structure and promoted emergence of EPS-overproducing mutants.

The spatial structure of the environment determines the invasion of mutations responsible for the excessive synthesis of EPS. To understand the feedback loop between ecology and evolution, however, one must understand the mechanisms underlying the structuring of the environment. Recent research (11) indicates that biofilm formation at the ALI influences the spatial distribution of diffusible metabolites produced at the ALI, in part through hydrodynamic instability-induced mixing. For instance, this mixing from the top to the bottom of the flask may explain why oxidized resazurin (pink dye) is visible so deeply under the ALI in Fig. 2C of the article (1), below the typical penetration length of oxygen from the ALI as observed by Koza (9).

This research shows that mutants who overproduce EPS benefit from a spatially heterogeneous and more concentrated oxygen distribution at the ALI because their redox state is well balanced there. Yet, it is still unclear why these mutants make up only a fraction of 30% of the population within a few millimeters of the ALI (Fig. 2C of the article (1)). The mechanisms that allow the stable coexistence of mutants and ancestors at the ALI are still unknown. Addressing this complex topic will undoubtedly require a collaborative effort from experts in microbial physiology, evolutionary biology, cell mechanics, hydrodynamics, and statistical physics.
